# Retraction: Structural, morphological, electrical, and dielectric properties of Na_2_Cu_5_(Si_2_O_7_)_2_ for ASSIBs

**DOI:** 10.1039/d5ra90057c

**Published:** 2025-05-14

**Authors:** Mohamed Ben Bechir, Mehdi Akermi

**Affiliations:** a Laboratory of Spectroscopic and Optical Characterization of Materials (LaSCOM), Faculty of Sciences, University of Sfax BP1171 3000 Sfax Tunisia mohamedbenbechir@hotmail.com; b Department Physics, College of Sciences, Jazan University P. O. Box. 114 Jazan 45142 Kingdom of Saudi Arabia makermi@jazanu.edu.sa; c Laboratory of Interfaces and Advanced Materials, Faculty of Science, Boulevard of the Environment, University of Monastir 5019 Monastir Tunisia

## Abstract

Retraction of ‘Structural, morphological, electrical, and dielectric properties of Na_2_Cu_5_(Si_2_O_7_)_2_ for ASSIBs’ by Mohamed Ben Bechir *et al.*, *RSC Adv.*, 2024, **14**, 9228–9242, https://doi.org/10.1039/D4RA01454E.

The Royal Society of Chemistry, with the agreement of the named author, hereby wholly retracts this *RSC Advances* article due to concerns with the reliability of the data.

There are concerns with the reliability of the application of the bond valence sum (BVS) method in this study. The authors acknowledge that their use of the method was based on crystallographic data obtained from VESTA, based on the published CIF file for Na_2_Cu_5_(Si_2_O_7_)_2_ using the similar compound ‘Li_2_Cu_5_(Si_2_O_7_)_2_’ as a reference,^1^ rather than independent experimental measurements, which may have led to inaccuracies in the analysis. The authors admit to a misapplication that stemmed from a misunderstanding of the requirements for precise and independent measurements necessary for BVS analysis and requested a correction.

Concerns have been raised about the reliability of the neutron powder diffraction (NPD) measurements. The authors acknowledge that the NPD measurements were conducted externally, and the facility where the measurements were performed was not explicitly acknowledged in the original article. The authors have not been able to provide the raw data for these measurements, and therefore we are not able to confirm their reliability.

In addition, the authors should have cited the work of Chikara *et al.*, as their research was inspired by previous studies on the similar compound Li_2_Cu_5_(Si_2_O_7_)_2_.^1^

Given the significance of these concerns, the Editor has lost confidence that the findings presented in this paper are reliable.

This Retraction supersedes the information provided in the Expression of concern related to this article.

The authors were informed about the retraction of the article. Mohamed Ben Bechir has agreed with the decision, the other author has not responded.

Signed: Mohamed Ben Bechir

Date: 6th May 2025

Retraction endorsed by Laura Fisher, Executive Editor, *RSC Advances*
